# A mobile cesarean birth center as a solution to improve access to surgical birth in rural Ethiopia: a mixed methods research protocol

**DOI:** 10.1186/s40814-021-00955-4

**Published:** 2021-12-15

**Authors:** Margo S. Harrison, Teklemariam Yarinbab, Brooke Dorsey-Holliman, Gregory A. Aarons, Ana Pilar Betran, Robert L. Goldenberg, Margaret Muldrow

**Affiliations:** 1 Department of Obstetrics & Gynecology, University of Colorado, Mail Stop B198-2, Academic Office 1, 12631 E. 17th Avenue, Rm 4211, Aurora, CO 80045 USA; 2 Mizan Tepi University Teaching Hospital, Mizan Aman, Ethiopia; 3 University of Colorado Adult & Child Consortium for Research and Delivery Science, Aurora, CO USA; 4 University of California San Diego, La Jolla, CA USA; 5 UNDP/UNFPA/UNICEF/WHO/World Bank Special Programme of Research, Development and Research Training in Human Reproduction (HRP), Department of Sexual and Reproductive Health and Research, World Health Organization Human Reproduction Programme, Geneva, Switzerland; 6 Columbia University Medical Center, New York, NY USA; 7 Village Health Partnership, Denver, CO USA

**Keywords:** Mixed methods, Qualitative methods, Cesarean birth, Mobile surgical center, Ethiopia, EPIS framework

## Abstract

**Background:**

As an evidence-based intervention to prevent maternal and neonatal morbidity and mortality, cesarean birth at rates of under 2%, which is the case in rural Southwest Ethiopia, is an unacceptable public health problem and represents an important disparity in the use of this life-saving treatment compared to more developed regions. The objective of this study is to explore an innovative clinical solution (a mobile cesarean birth center) to low cesarean birth rates resulting from the Three Delays to emergency obstetric care in isolated and underserved regions of Ethiopia, and the world.

**Methods:**

We will use mixed but primarily qualitative methods to explore and prepare the mobile cesarean birth center for subsequent implementation in communities in Bench Sheko and West Omo Zones. This will involve interviews and focus groups with key stakeholders and retreat settings for user-centered design activities. We will present stakeholders with a prototype surgical truck that will help them conceive of the cesarean birth center concept and discuss implementation issues related to staffing, supplies, referral patterns, pre- and post-operative care, and relationship to locations for vaginal birth.

**Discussion:**

Completion of our study aims will allow us to describe participants’ perceptions about barriers and facilitators to cesarean birth and their attitudes regarding the appropriateness, acceptability, and feasibility of a mobile cesarean birth center as a solution. It will also result in a specific, measurable, attainable, relevant, and timely (SMART) implementation blueprint(s), with implementation strategies defined, as well as recruitment plans identified. This will include the development of a logic model and process map, a timeline for implementation with strategies selected that will guide implementation, and additional adaptation/adjustment of the mobile center to ensure fit for the communities of interest.

**Trial registration:**

There is no healthcare intervention on human participants occurring as part of this research, so the study has not been registered.

## Administrative information

Protocol Version: 1.0

Type of Article: Study Protocol

Funding: Research reported in this publication was supported by the Eunice Kennedy Shriver National Institute of Child Health & Human Development of the National Institutes of Health under Award Number R21HD102720 & 5K12HD001271. The content is solely the responsibility of the authors and does not necessarily represent the official views of the National Institutes of Health.

Study Sponsor:

National Institutes of Child Health and Human Development

Phone: 1-800-370-2943

Email: NICHDInformationResourceCenter@mail.nih.gov

Fax: 1-866-760-5947

Mail: P.O. Box 3006, Rockville, MD 20847

Role of Study Sponsor: The National Institutes of Child Health and Human Development had no role in the study design or writing of the protocol, or the decision to submit the protocol for publication.

Study Oversight: As this is not a clinical trial there is no coordinating steering committee, endpoint adjudication committee, or data management team.

## Background

### Background and rationale

When medically indicated and properly conducted, cesarean birth saves maternal, fetal, and neonatal lives [1–3]. Historically, the World Health Organization (WHO) recommends that a 10–15% cesarean birth rate is appropriate among all global populations [3]. Although the exact ideal cesarean section (CS) rate is unknown, CS rates at a population level between 9 and 19% have been associated with reductions in mortality [3–5]. As an evidence-based intervention to prevent maternal and neonatal morbidity and mortality, cesarean birth at rates of under 2%, which is the case in rural Southwest Ethiopia, is an unacceptable public health problem and represents an important disparity in the use of this life-saving treatment compared to more developed regions.

Low cesarean birth rates plague many regions of sub-Saharan Africa, so the context of rural Southwest Ethiopia is generalizable to other settings [6]. Barriers to cesarean birth as an intervention to prevent morbidity and mortality in sub-Saharan Africa include those described by the Three Delays model: (1) the delay in the decision to seek care, (2) the delay in reaching appropriate emergency obstetrical care, and (3) the delay in receiving adequate care when the facility is reached [7]. Preliminary data from our target community in Southwest Ethiopia found that the Three Delays is representative of barriers to accessing cesarean birth; delays made it “virtually impossible” for many women to reach the hospital [8, 9].

The Three Delays model describes delays to emergency obstetric care and was published 25 years ago, but little to no progress has been made in addressing these delays in many locations and it is still relevant as a barrier to progress in the field [7]. In a survey of 389 women living in our study region, delays were experienced by 76% of respondents [8]. On average, the women reported that it took 5 h to reach a facility (the second delay), with 96% of respondents reporting long wait times at the facility (the third delay) [8]. The Ethiopian pregnancy-related mortality ratio is 412 deaths per 100,000 live births [10]. The leading cause of maternal and perinatal death is obstructed labor, which can be resolved with a cesarean birth [9, 11–16]. Therefore, there is a critical need for innovative solutions to low cesarean birth rates resulting from the Three Delays to emergency obstetric care in isolated and underserved regions of Ethiopia and the world [8, 17].

### Mobile cesarean birth center and prior research

Médecins Sans Frontières, a clinical aid organization, has been providing cesarean birth in tents in conflict zones in low- and middle-income countries [18]. In 2017, a quarter of the surgeries (35,000) they performed were cesarean births [18]. This recent prior experience suggests that mobile cesarean birth centers may be a safe and effective method of delivering obstetric care. Mobile surgical trucks have been successfully used in Latin America to deliver surgery (including gynecologic procedures) under similar conditions, and Médecins Sans Frontières provided 35,000 cesarean births in mobile, temporary tents in 2017 in low-resource settings [18–21]. Prior to their success, surgical trucks were used for 15 years in Ecuador to deliver surgical services to remote areas of the country [19–21]. The Ecuadorian truck was outfitted with an operating table, surgical light, anesthesia machine, cautery, laparoscopy, and a scrub sink and changing area [19]. It was air conditioned and had its own water supply [19]. Of 4545 surgeries performed in the truck, 651 (14.3%) were gynecologic [19]. There were reportedly no deaths, four major complications (cardiac arrest, pulmonary embolism, gastrointestinal injury, transfusion), and three minor complications (two wound infections and one laparotomy conversion). These rates are lower than complication rates reported after general surgery [19, 22].

A “Hospitainer” is a customizable shipping container that has been outfitted to provide medical and surgical care; it includes an operating room, a pre- and post-operation room, a storage/sterilization room, medical equipment, consumables, a generator, and medicines for surgical procedures [23]. Required specifications of such units include detail on climate control, clean water supply, that all equipment should be battery powered but rechargeable, and ideally the vehicle should be parked for procedures [23, 24]. Hospitainer reports that one 800 l water and one 800 l waste tank are included with the container with each requiring refreshing/emptying about every week. We will use this Hospitainer as our proposed prototype (picture below; provided with consent of Hospitainer), presenting a concrete example that will give stakeholders a model to consider as they explore, design (adapt), and prepare the unit to meet their needs. These Hospitainers have already been used to provide emergency obstetrical care in Sudan, Congo, Sierra Leone, Somalia, and Gabon with success (as well as in other regions, including the Middle East, Europe, and Americas).

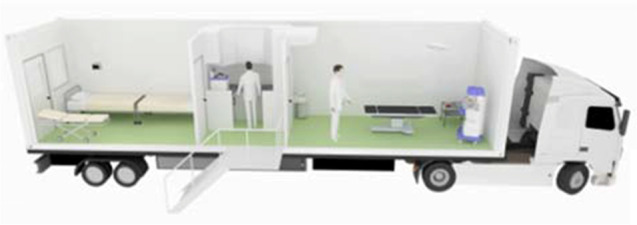



In terms of staffing such a mobile cesarean birth center, Ethiopia has increased surgical staff nationally through task-shifting emergency surgery to non-physician providers called Integrated Emergency and Surgical Officers (IESOs) [15, 25–29]. The program was introduced in 2009; IESOs pursue a 3-year course in emergency obstetrics and general surgery, with Helping Babies Breathe and newborn resuscitation as integral components of the training [15, 30]. From 2012 to 2014, 4075 operations were performed by IESOs, 63% of which were cesarean births [15]. During this timeframe, the cesarean birth rate was 12.5% [3, 4, 15, 31]. However, IESOs work primarily at referral facilities, which highlights the lack of accessible, high-quality, facility-based surgical care and the need for mobile units that still exist in rural areas despite their training. Until healthcare facilities in rural areas can provide cesarean birth, we propose that non-physician surgeons might be able to provide services in mobile units such as the Hospitainer.

## Methods/design

### Scientific premise

Despite extensive research to improve access to care in low- and middle-income countries (LMIC), access to cesarean section to all women in need is not universal, even though the Three Delays model was published 25 years ago [7]. As such, there is a critical implementation gap in determining how best to provide cesarean birth in vast, rural regions of the continent. Our overarching hypotheses are (1) the cesarean birth surgical disparity in rural Ethiopia can be addressed by the implementation of a novel, mobile cesarean birth center staffed by advanced practice providers and (2) the methods we will use to explore, prepare, and design the center for eventual adaptation, implementation, and dissemination will be generalizable to other underserved settings and/or surgical disparities*.* Preliminary data from 21 individuals from target communities and 10 physicians at the regional referral center found that the mobile cesarean birth center concept was highly (90–100%) acceptable, appropriate, feasible, and usable in their setting. Therefore, our overall objective is to adapt a mobile cesarean birth concept for use in geographically isolated and underserved areas of Ethiopia in order to prepare for subsequent implementation and potential dissemination, considering all three delays. We want to learn about the acceptability, appropriateness, and feasibility of the mobile unit as a semi-permanent solution for access to care, which will eventually be replaced by improved healthcare infrastructure in the rural areas. We will collect preliminary data on cost considerations as well as the mobile versus stationary nature of the potential unit.

### Specific objectives

Our multidisciplinary team of cesarean birth and obstetrics, implementation science, and public health experts as well as representative stakeholders from Ethiopia are poised to successfully achieve our objectives, guided by the Exploration-Preparation-Implementation-Sustainment (EPIS) framework (implementation process framework), as follows: [32, 33]

Objective 1: EXPLORE (first phase of the EPIS framework) the outer and inner contexts of the communities in rural Ethiopia where we will study the pre-implementation of the mobile cesarean birth center [33]

Implementation strategies: Identify barriers and facilitators to the delivery of cesarean and test the appropriateness, acceptability, and feasibility of a mobile cesarean birth center as one potential solution [34]

Objective 2: PREPARE (second phase of the EPIS framework) to address the outer and inner contextual components of the communities where we will study the pre-implementation of the mobile cesarean birth center [33]

Implementation strategies: Develop a formal implementation blueprint (with implementation strategies) for the center that addresses the barriers and facilitators (including all Three Delays) to the delivery of cesarean birth [34]

### Research frameworks

In order to adapt and prepare the mobile cesarean birth center to address the public health problem of low cesarean birth rates in the region, our research is guided by the EPIS implementation research process and determinant framework; the author of the framework is part of our research team and has applied the framework in sub-Saharan Africa, previously [14, 32, 33, 35–37]. EPIS provides guidance on understanding barriers and facilitators and adapting our intervention (exploration phase, aim 1); taking what is learned in exploration and preparing to implement it (preparation phase, aim 1); putting structures, processes, and action into place (implementation phase, aim 2); and beginning with the end goal in mind (sustainment phase) so that implementation gains are realized and have the greatest public health impact [14]. The framework examines both the “Outer Context,” which refers to system-level factors in the country and community, and “Inner Context,” which refers to organizational level factors, and “Bridging Factors” that link outer and inner contexts (e.g., policies, collaborations), inter-organizational relationships, and innovation factors (e.g., cesarean procedures in Hospitainers) [32, 33, 38].

Using a stakeholder framework, we will include patients and the public, providers, purchasers, payers, policy-makers, product makers, and principal investigators [39]. In our target region, this translates to women, husbands, and community leaders (patients and the public); IESOs/physicians (providers); Ministry of Health and Local, Zonal, Regional, and Federal Government representatives (purchasers, payers, policy-makers); and our research collaborators (principal investigators). These will be the stakeholders involved in our research activities, guided by the EPIS framework, to ensure rigorous execution of our objectives.

### Study setting

The only referral facility capable of providing cesarean birth for the Bench Sheko and West Omo Zones of Ethiopia is Mizan-Tepi University Teaching Hospital (MTUTH), which is located in the Mizan-Aman, Ethiopia, in the Southern Nations Nationalities and People’s Region (SNNPR). The catchment area of the hospital includes 2.5 million people in the Bench Sheko and West Omo Zones. Women account for 51% of the population, and 48% are of reproductive age; this suggests that about 612,000 women of reproductive age live in these two zones [10]. Based on the overall population size, the WHO would recommend five emergency obstetric facilities in the region [40]. If it is assumed that half are pregnant with a 10% cesarean birth rate, about 30,000 women per year would require cesarean birth. This would require MTUTH and four other “Hospitainers” to perform about 6000 cesareans per year, which is 16–23 cesareans per day, or one surgery every 1–1.5 h in the unit. A cesarean takes about 30–60 min to perform, which leaves 30–60 min to clean before the next birth. Per personal communication with Hospitainer, they estimate their containers are capable of 8–10 surgeries in 10 h.

### Community participants

We estimate a need for four Hospitainers in the region to meet emergency obstetrical care WHO guidelines [40]. We intend for these four containers to initially serve the needs of at least four tribes in five communities: the Bench, Me’en, Dizy, and Suri tribes. Because each tribal group is unique, this population includes significant heterogeneity. We visited all of these communities with our partners in April 2019 to discuss the project and begin the necessary preparations for recruitment; the concept for the mobile cesarean birth center was enthusiastically received at that time.

#### Sample size

We will recruit volunteers in the communities with the help of community leadership until we have at least 10 participants in each morning and afternoon session, although we expect numbers closer to 20 participants per session. At MTUTH, we will invite all physicians and administrators to participate, as well as Zonal health leadership [41].

#### Recruitment

##### Women of reproductive age

Women 18 years and older seeking antenatal or postnatal care at the community health clinics will be offered enrollment, in person, by study staff. We will offer enrollment until 10 women agree to participate.

##### Men

Community (religious or tribal) leaders will offer men enrollment in person at community gatherings that they host, until we have at least 10 participants.

##### Community leaders

Dr. Muldrow, President of 501c3 Village Health Partnership (our research partner), has a 40-year relationship with leaders in our target communities. By leveraging both her established relationships and discussing the specific project during our recent travel to the sites in 2019, we have started prep-to-research activities. Community leaders are generally tribal leaders, religious leaders, or other prestigious members of the community; in order to enter communities during our visit, we had to engage these individuals through Dr. Muldrow’s established partnerships. These are the most crucial stakeholders to engage, in our experience and according to the physicians at MTUTH as presented in the preliminary data section.

##### Clinicians/administrators/health officials

We have already conducted preliminary assessments of the health centers in our target communities and as such have met with the aforementioned stakeholders, including some of the zonal and woreda (regional) health officials.

### Study design

For objective 1, we will conduct semi-structured interviews and focus groups with representative stakeholders (community members and leaders, clinicians, administrators); participants will discuss the center and alternative solutions and ways to ensure the solution(s) respond to the Three Delays and meet community needs [42]. To achieve objective 2, representative stakeholders in modeling and simulating the proposed cesarean birth center [or other potential solution(s)], we will conduct cyclic consensus discussions to optimize the cesarean birth center into a clinically implementable innovation that will be adaptable for future dissemination [43]

To achieve our objectives, we will host five, 2-day retreats, one with each of the Bench, Me’en, Dizzy, and Suri communities at a location chosen by community leadership, and a sixth retreat at MTUTH with the purchasers/payers/policy-makers/providers contingent of outer and inner context stakeholders. The exploration phase of the EPIS framework begins when stakeholders are aware of a public health need and are considering ways to address it [8]. Providers at MTUTH were surveyed and reported that the three delays are relevant to care for their patients, and preliminary data we collected from patients at the facility who hailed from all areas of the zone (we did not collect their self-identified tribal group so this data may not reflect our study population) suggested that women and their husbands are also aware of the need for improved access to emergency obstetric care and consider the mobile cesarean birth center as an acceptable, appropriate, and feasible solution [17].

#### Study activities, objective 1, exploration phase

To achieve the first objective, the exploration phase, we will focus on exploring the Hospitainer for pilot testing in the region. To do this, on the first day of the visit, we will have a morning session with women and an afternoon session with men and community leaders as most community leaders are male (female leaders will participate with the female focus groups); each iteration noted in the table (Table 1) will take about 45 min to complete. The goal of these retreats is to ensure that the Hospitainer is explored to address specific barriers and facilitators to emergency obstetric care in each community, using Human-Centered Design methods and considering EPIS constructs [43–45]. Human-Centered Design “offers problem solvers…a chance to design with communities, to deeply understand the people they’re looking to serve…and to create innovative new solutions rooted in people’s actual needs” [43–45]. These methods have been used successfully in rural Ethiopia with teff (grain) farmers to co-create interventions that have been designed through academic-community collaboration, but it is innovative to apply them to optimizing a surgical intervention for cesarean birth, as outlined in Table 1 [43, 44].

**Table 1:** Exploration phase: co-creation retreat

	Activity	Method	Goal
Iteration 1	Co-creation session	Role playing: act out a woman and her husband/family using the cesarean birth center • One group; act out the scenario • Discuss ideas	What was realistic? What worked? What issues arose?
Iteration 2	Co-creation cession	Storyboard: visually plot out the elements of the cesarean birth center • Two groups; draw the story • Present ideas	Who will use it? Where will they use it? How will they use it?
Iteration 3	Co-creation session	Individual optimization: ask each participant to write down/draw what they would change about the storyboard they worked on • Individual work time • Present ideas	Make it efficient Make it effective Make it appropriate Make it acceptable
Iteration 4	Co-creation session	Business Model Canvas: complete a worksheet about key aspects of the cesarean birth center and what key criteria are required for the cesarean birth center to provide high-quality specialized care for obstructed labor • Two groups; complete worksheet; present ideas	Make it feasible Identify outcomes
Iteration 5	Co-creation session	Integrate feedback and iterate: share the feedback, synthesize, refine idea • Focus group format, guided • **Conduct Four-Item quantitative assessment tool** [46]	Ensure it is appropriate Ensure it is acceptable Ensure it is feasible

#### Study activities, objective 2, preparation phase

To achieve our second objective, in the preparation phase, we will produce a detailed implementation plan to capitalize on implementation facilitators and address potential barriers and further assess needs for adaptation [27, 47]. Critical to this phase is planning implementation strategies and developing a positive implementation climate in which the adapted Hospitainer is valued and supported; in order to achieve this goal, during the exploration phase (Table 1) and preparation activities (Table 2), the service and policy environment and the characteristics of the individuals (women who are patients and consumers) who will use the Hospitainer must be clarified [47]. In order to consider the inter-organizational relationships between entities such as governments, funders, professional societies, and consumers, the second day of the retreat will focus on taking the explored and preliminarily adapted Hospitainer approach that stakeholders have determined is appropriate, acceptable, and feasible, and develop a specific, measurable, attainable, relevant, and timely implementation blueprint(s), with implementation strategies defined, through activities listed in Table 2 [32, 33, 47].

**Table 2:** Preparation phase: develop the implementation blueprint(s) and strategies

	Activity	Method	Goal
Step 1	Live prototype	Stand in the community space where the cesarean birth center will be piloted: stress test (role play) the solution in “real world conditions” • Walk through use case scenarios including people trying to use the Hospitainer for non-obstetric purposes; troubleshoot obstacles that arise	Sort logistics of use Understand feasibility Understand viability
Step 2	Resource assessment	Resource assessment: complete a worksheet about what exactly is needed to execute the solution including issues like water, electricity, and laundry • Two groups; complete worksheet; present ideas	Distribution, activities Capabilities, responsibilities
Step 3	Build partnerships	Relying on partners: who do you need to make implementation happen and what implementation strategies will be necessary • Focus group format, guided	Make it feasible Select implementation Strategies
Step 4	Roadmap	Create an implementation plan: timeline, responsibilities, set benchmarks on a large calendar, process map, produce a logic model • Focus group format, guided	Purchasing, delivery Supply chain

#### Data collection methods

At the beginning of the retreats, verbal consent will be obtained from participants and sociodemographic information will be collected. The sessions will be recorded and transcribed in Amharic. Translation into English will be performed by the facilitators (analysts).

#### Data management

No identifiable information will be collected in the proposed research project. There will be no biospecimens or other records obtained. De-identified transcribed data will then be transmitted securely in English and stored on password-protected computers at the University of Colorado under a data transfer agreement defined in a memorandum of understanding. These data will not be linked to any other previously collected data.

### Outcomes

Completion of objective 1, or the exploration phase, will allow us to describe participants’ perceptions about barriers and facilitators to cesarean birth and their attitudes regarding the appropriateness, acceptability, and feasibility of the Hospitainer as a solution. Each community may have variants in the prototype; given the eventual plan for four Hospitainers, tribes will be able to adapt the unit per their preferences (external color, decorations). This approach is consistent with work in the adaptation of evidence-based practices while preserving core elements (e.g., surgical setting and procedures) while making adaptations to fit local culture and preferences [48]. Observing, addressing, and documenting these adaptations may assist with subsequent dissemination to other local or global regions.

Completion of objective 2, or the preparation phase, should result in a specific, measurable, attainable, relevant, and timely (SMART) implementation blueprint(s), with implementation strategies defined, as well as recruitment plans identified [32, 33, 47]. This will include the development of a logic model and process map, a timeline for implementation with strategies selected that will guide implementation, and additional adaptation/adjustment of the Hospitainer to ensure fit for the communities of interest [34]. The implementation will be more successful if there is a high degree of fit between the values and needs of the stakeholders and the characteristics of the innovation to be implemented [27]. A summary of the outcomes is presented in Table 3.

**Table 3:** Outcome summary table

Phase	EPIS activity	Objective	Methods	Tools	Outcomes
*Exploration*	Consider emergent or existing need of patients, clients, or community	Understand context, barriers/facilitators, and Hospitainer as potential solution	Focus groups Quantitative assessment	EPIS and Human-Center Design-framed interview guide Weiner et al. Tool [46]	Barriers and facilitators Acceptability Appropriateness Feasibility
*Preparation*	Assess the need for adaptation and develop a detailed implementation plan	Conduct formative research to inform implementation and design Hospitainer tailored to local context	Focus groups	EPIS and Human-Center Design-framed interview guide	Logic model Process map SMART timeline Implementation strategies *Plan recruitment*

### Analytic methods

We will utilize qualitative content analysis to analyze the data [49]. Using an inductive approach, the team will develop a set of codes from multiple readings of the transcripts using Atlas.ti qualitative data management software [50]. The senior professional research assistant will code the transcripts with the PI and qualitative expert heavily involved in codebook development (e.g., coding the first few iterations), with feedback and participation of the facilitators. All discrepancies in the code definitions and applications will be reconciled through consensus. Codes will be clustered into related categories, which will then guide theme development [42]. We will utilize a quantitative assessment tool to have participants rate the appropriateness, acceptability, and feasibility of the intervention during iteration 5 (Table 1) [46]. The tool uses four questions to ask about each concept with a grading system to quantify the results [46]. It will be translated, back-translated, and piloted to ensure applicability to the study populations. Qualitative research with community members has been successful in Ethiopia, previously, including in this region and regarding barriers to surgical care, specifically [8, 51–57]. The two types of data will be triangulated to produce a joint display of our qualitative and quantitative findings. The same analytic methods that were used during the exploration phase will be used during the preparation phase.

### Participant timeline

All our study activities are planned for October 2021 and will not require any ongoing follow-up of study participants.

## Discussion

### Harms

Risks to our study subjects include loss of privacy and confidentiality related to participating in any of the focus groups or retreats. For participants who choose to participate with other members of their community, there may be psychological, social, and potentially cultural risks associated with engaging in study discussions. We will inform participants that they may leave the focus group at any time if they feel uncomfortable and will begin the sessions with setting ground rules in an effort to create an environment of safety and trust. It is also possible that there could be a breach of study data during secure transmission or storage of our transcribed and translated data; however, these transcripts will not include any identifying data. If any identifying data was accidentally collected during the interview, it will not be included in the transcript. We will inform participants of these risks prior to verbal consent, and if they do not wish to participate, they can decline involvement in the study; however, these risks are highly unlikely. No alternative treatments or procedures are relevant in the context of this study; patients can decline to participate, which is their only alternative to consenting to participate in study activities. We plan to collect and report any adverse events or unintended effects that arise during data collection as observed by study staff.

### Protection again harms

The planned strategy for protecting against and minimizing all potential risks identified is to properly explain the social nature of focus groups to participants to help them understand they will be providing information publicly, in front of other community members and peers. Helping them to fully understand what focus groups are may assist them in deciding whether or not they want to be part of it and be exposed to potential psychological, social, and cultural risks that may exist. Additionally, in terms of the transfer of de-identified data, this will occur securely over an encrypted connection directly to CU whose password-protected servers and networks can only be accessed by employees with access to the system. Even if the data were to be breached, given that it is encrypted and transmitted in a de-identified format, there is less of a chance of that data being directly attributed to any given individual. As we do have physicians performing the consent and the study activities, if they determine that a patient has incurred an adverse outcome related to the study activities, they will be able to manage the logistics of getting that patient to appropriate care in such an event.

### Auditing

No audits are planned at this time as the principal investigator will be present to oversee all study activities as data is being collected.

### Dissemination policy

The investigators plan to communicate study results to participating professionals and the communities visited by presentation of a report and accompanying PowerPoint to be emailed to community and facility leadership. There is no intention to use professional writers to convey study results, and authorship eligibility guidelines will reflect those required by journal submission. There are no plans for granting public access to the participant-level dataset.

### Protocol amendments

No protocol amendments have yet been made, but if they do occur, changes to eligibility criteria, outcomes, and analyses will be communicated to COMIRB and MTUTH as required by their approvals.

## Data Availability

The data has not yet been collected, but a data use agreement will be formally signed allowing all principal investigators (Harrison, Muldrow, Yarinbab) complete access to the data with provisions that all investigators need to review any analyses prior to submission for publication.

## Abbreviations

SMART: Specific, measurable, attainable, relevant, and timely
WHO: World Health Organization
CS: Cesarean section
IESOs: Integrated Emergency and Surgical Officers
LMIC: Low- and middle-income countries
EPIS: Exploration-Preparation-Implementation-Sustainment
MTUTH: Mizan-Tepi University Teaching Hospital
SNNPR: Southern Nations Nationalities and People’s Region
